# Redesigning Care of Hospitalized Young Adults With Chronic Childhood-Onset Disease

**DOI:** 10.7759/cureus.27898

**Published:** 2022-08-11

**Authors:** Colby D Feeney, Alyssa Platt, Jesse Rhodes, Yasmin Marcantonio, Sonya Patel-Nguyen, Tyler White, Jonathan A Wilson, Jane Pendergast, David Y Ming

**Affiliations:** 1 Hospital Medicine, Duke University Health System, Durham, USA; 2 Medicine and Pediatrics, Duke University School of Medicine, Durham, USA; 3 Biostatistics and Bioinformatics, Duke University School of Medicine, Durham, USA; 4 Medicine, Duke University School of Medicine, Durham, USA; 5 Performance Services, Duke University Health System, Durham, USA; 6 Medicine, Pediatrics, and Population Health Sciences, Duke University School of Medicine, Durham, USA

**Keywords:** med-peds, care redesign, chronic disease, adolescent, inpatient medicine, hospital medicine, young adult, transition

## Abstract

Background

Young adults with chronic childhood-onset disease (CCOD) are routinely admitted to internal medicine hospitalist services, yet most lack transition preparation to adult care. Providers and patients feel the strain of admissions to adult services in part due to their medical and social complexity.

Methods

We performed a descriptive study of a care redesign project for young adults with CCOD hospitalized at a large, tertiary care academic hospital. We describe the process of implementation of the Med-Peds (MP) service line and characterize patients cared for by the service. We measured and analyzed patient demographics, process implementation, healthcare screening, and healthcare utilization data.

Results

During the 16 months of the study period, 254 patients were cared for by the MP service line, accounting for 385 hospitalizations. The most common CCODs were sickle cell disease (22.4%) and type 1 diabetes (14.6%). The majority (76%) of patients completed transition readiness assessment, and 38.6% completed social determinant of health (SDH) screening during their admission. Patients had high prevalence of SDH with 66.7% having an unmet social need. The average length of stay was 6.6 days and the average 30-day readmission rate was 20.0%.

Conclusions

There is opportunity to redesign the inpatient care of young adult patients with CCOD. The MP service line is a care model that can be integrated into existing hospital medicine teams with MP physicians. Hospitals should consider redesigning care for young adults with CCOD to meet the transitional and social needs unique to this patient population.

## Introduction

Each year an estimated 750,000 children with special healthcare needs enter adulthood and transfer to adult-oriented healthcare [1]. Despite best practice recommendations calling for years of preparation to successfully transition to adult-oriented healthcare, only 17% of youth with special healthcare needs have received any transition preparation [2]. Studies suggest that adult hospitalists may not be comfortable caring for young adults with chronic childhood-onset disease (CCOD), such as sickle cell disease and cystic fibrosis, due to unfamiliar, complex medical illnesses, and various unmet social needs [3,4].

The young adult years for patients with CCOD are often marked by worsening health, unstable social structures, and an increase in emergency department visits and hospitalizations [4-9]. For young adults with CCOD, hospitalization can be a particularly negative experience. Young adults with CCOD identify several factors contributing to this, including parental exclusion from decision-making, unfulfilled care needs, and perceived lack of understanding of the impact of the hospitalization on the patient's overall health [10].

As there is a growing number of young adults with CCOD who will require hospitalization, adult inpatient providers must be prepared to care for them [1,11]. Inpatient providers should have the knowledge and skills to help usher these patients into adult-oriented care in order to create a more positive inpatient experience while providing high-quality care. Hospitalization is an opportune time to identify and begin to address their medical, social, and transition-specific needs. Despite this clear opportunity, there is a lack of research to study the effects of inpatient care redesign for this population. To address these critical gaps, we designed and implemented an inpatient med-peds (MP) service line to care for young adults with CCOD within the existing framework of general medicine. This study aims to describe the care redesign and implementation of an inpatient hospitalist service line focused on transition needs, and to characterize our population of young adults with CCOD hospitalized on the MP service line.

## Materials and methods

Setting

We implemented the MP service line in June 2019 within the general medicine service at a large tertiary care academic hospital, Duke University Hospital, Durham, North Carolina, United States, with more than 900 inpatient beds. Children less than 18 years of age are admitted to the tertiary children’s hospital consisting of 190 beds located within the main hospital. The majority of hospitalized patients 18 years of age and older are admitted to the general medicine service in the main hospital. In 2019, there were approximately 7,500 discharges from the general medicine service, of which 48% were managed by hospitalist physicians and advanced providers. Of the 31 hospital medicine physicians, five physicians who had completed combined internal medicine and pediatrics residency training programs rounded on the MP service line.

Planning

We measured the number of young adults with CCOD in general medicine through chart review and the use of a self-service reporting tool, SlicerDicer©, within our electronic health record (EHR) (Epic Systems Corporation, Verona, Wisconsin, United States). Chart review and EHR data abstraction informed a planning estimate of 10-12 inpatients eligible for the MP service line per day. Subsequently, we met with key stakeholders to co-design service line criteria, workflows, and interventions for approximately six months prior to the start of the service. Stakeholders included the future rounding hospitalists on the service, hospital medicine leadership, case management, and outpatient experts in transitional care.

Inclusion and exclusion criteria

Hospitalized general medicine patients were eligible for care on the MP service line if they met the following inclusion criteria: (1) 18 years of age or older, and (2) have a CCOD or sequela of CCOD causing ongoing functional limitations. CCODs were defined as chronic medical diagnoses that have onset before the age of 18, including congenital conditions such as spina bifida and acquired conditions such as type 1 diabetes. Older patients (>30 years old) with CCOD were prioritized for the MP line if they had a developmental disability or high medical complexity as determined by the clinician.

Team structure and assignment

The MP service line team consisted of the MP hospitalist, a case manager, and a clinical assistant. The case manager functioned to identify discharge needs and assist with the coordination of outpatient services. The clinical assistant scheduled follow-up appointments and administered patient screening tools as described below. A hospitalist nurse or physician (triage hospitalist) whose sole responsibility was team assignment of general medicine patients assigned eligible patients to the service upon admission. The triage hospitalist and MP hospitalist shared in decision-making about patient team assignments as needed. Eligible patients were not placed on the line when there were census or capacity constraints. Patients were not geographically located.

Core components

In an effort to meet the unique needs of hospitalized young adults with CCOD, the MP service line had four components. First, MP hospitalists who had completed combined internal medicine-pediatrics residency training cared for the patients as the primary attending physician. Secondly, each patient’s transition readiness was evaluated using the 20-item transition readiness assessment questionnaire (TRAQ). Either the attending physician or clinical assistant administered the TRAQ, a validated tool to assess self-management and self-advocacy skills essential to successful, independent self-care [12]. TRAQ responses link to scores 1-5, which correlate to the Stages of Change continuum to describe the patient’s stage in behavior change from pre-contemplation (score of 1) to maintenance (score of 5) [13]. Responses were directly entered into the EHR. If the patient was dependent on a caregiver, the TRAQ was administered to the caregiver to assess the patient’s transition readiness. Based on responses, the attending physician provided specific transition education to the patient or caregiver.

Thirdly, social determinants of health (SDH) were assessed using screening questions in the EHR. SDH screening included standardized questions about financial strain, food insecurity, and transportation barriers [14]. SDH screening began in November 2019.

**Figure 1:**
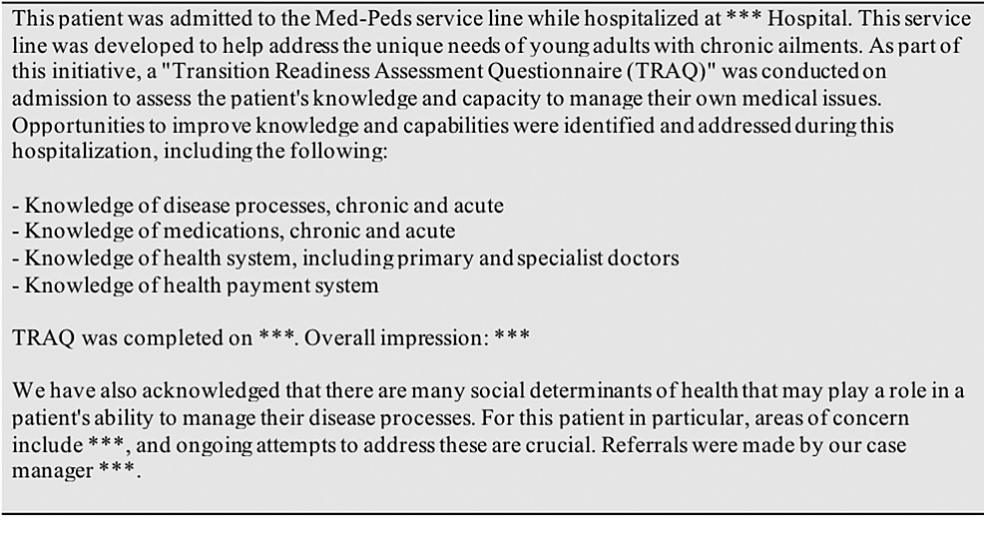
Discharge summary template

Finally, the MP service line attending physician coordinated care with outpatient providers at the time of discharge by scheduling follow-up appointments with primary care and specialty providers, arranging transfer to adult care services when needed, and highlighting transition and SDH needs for each patient in a customized discharge summary template (Figure 1).

Data extraction and analysis

Data summarizing hospitalizations at our hospital, demographic characteristics, and outpatient follow-up visits were extracted from EHR for patients cared for by the MP service line from June 29, 2019, through October 31, 2020. All data were obtained directly from the EHR with the exception of CCOD. The primary author performed a chart review to determine CCOD diagnosis and classify the disease into broader categories. A second MP line attending physician verified the assignment of CCOD by completing a chart review of 10% of the sample, which resulted in a 100% rate of agreement.

Patient demographics were extracted at the time of the first MP service line hospitalization. We calculated distance from the hospital as the geodetic distance between zip code centroids of the hospital and the patients’ reported home zip code in order to approximately identify the proportion of patients living near the hospital.

In order to summarize healthcare utilization prior to entry into the MP service line, hospitalization data were queried to include any prior inpatient admission at our hospital, regardless of whether the patient was cared for by the MP service. Hospital encounter level data included inpatient admissions on the MP service of all patients who had ever been cared for on the service during the study period. A 30-day rate of readmission was calculated by quantifying all subsequent admissions to our hospital in the 30 days following discharge, regardless of whether those admissions were on the MP service line.

SDH questions may be asked during any healthcare encounter. In order to quantify SDH completion as part of the MP service line, only SDH questionnaires completed during an MP hospitalization were counted towards the MP service questionnaire completion rate. In order to summarize SDH characteristics for our patients, the SDH questionnaires completed closest in time to the first MP service hospitalization were described, regardless of whether the SDH was completed during an MP service hospitalization. Positive responses to the financial strain question included answers of somewhat hard, hard, or very hard. Report of worrying about food ever was considered a positive response to food insecurity.

We utilized descriptive statistics to characterize patients cared for by the service line. We performed bivariate statistical testing to compare patient characteristics between those patients who had TRAQ and SDH questionnaires completed and those who did not in order to assess the generalizability of non-missing questionnaires. For those comparisons, we used Wilcoxon rank-sum tests to compare continuous covariates, Chi-square tests to compare categorical covariates with cell sizes greater than 10, and Fisher’s exact tests to compare categorical covariates with any cell size less than 10.

The Institutional Review Board of Duke University Health System, Durham, North Carolina, United States, approved this study (approval number: Pro00104906) and determined it exempt from full review as a quality improvement project.

## Results

Patient characteristics

**Figure 2:**
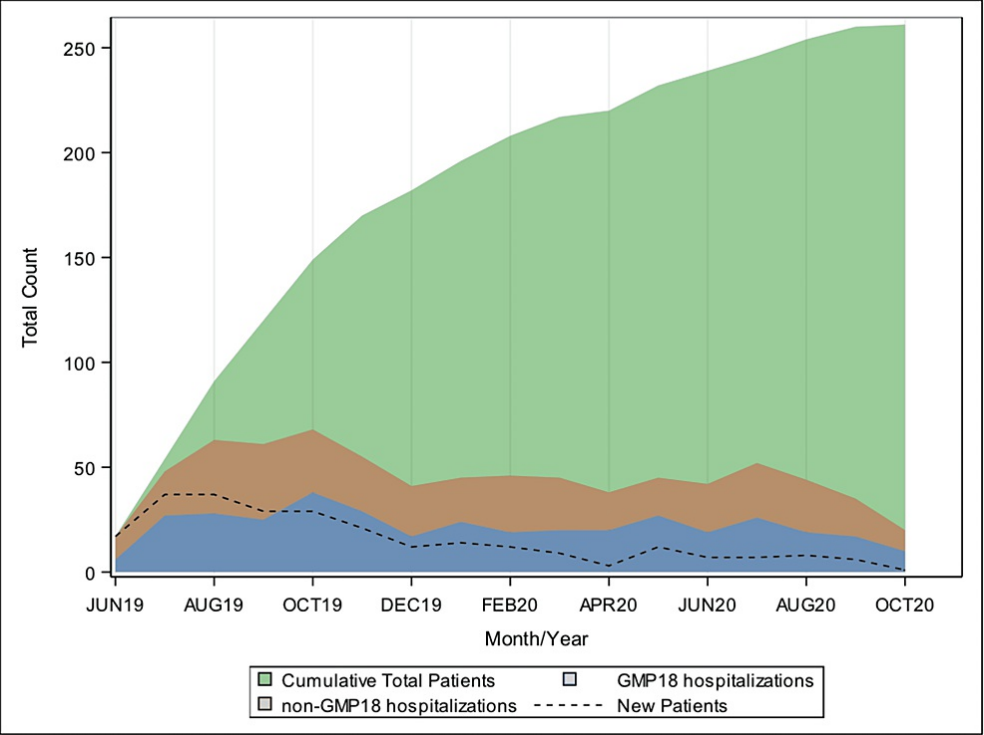
Patients’ first admission to the Med-Peds service line and hospitalizations over the study period (June 2019-October 2020) GMP18: Med-Peds service line; non-GMP18: any other service line

**Figure 3:**
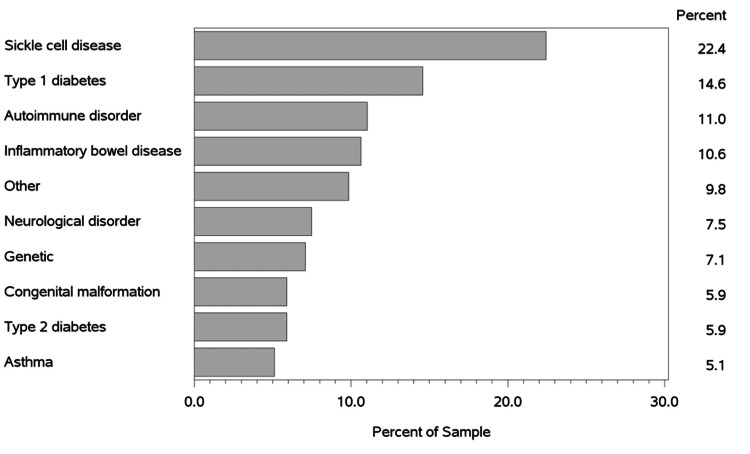
Chronic childhood-onset diseases (CCODs) of the patients on the Med-Peds service line

During the 16 months of the study period, 254 patients who met the inclusion criteria were cared for on the MP line during an inpatient stay, with 70% of the patients being added in the first six months of the service line (Figure 2). The most frequently managed CCODs were sickle cell disease (n=57, 22.4%) and type 1 diabetes (n=37, 14.6%) (Figure 3). The median patient age at entry into the service line was 26 years (IQR: 21-31). The majority of patients self-identified as Black or African American (n=143, 56.3%), and of non-Hispanic ethnicity (n=235, 92.5%). A majority of patients (n=178, 70.1%) lived more than 15 miles away from the hospital. Upon entry into the MP service line, the majority of patients had public insurance (n=143, 56.3%), of which 65% had Medicaid and 32.2% had Medicare or Medicare Advantage. Most identified a primary care provider (PCP) (n=217, 85.4%). Approximately half of the patients (51.2%) had no hospitalizations at our health system hospitals in the prior two years. Those who had prior hospitalizations in the previous two years were evenly split between those with one to three prior stays (n=60, 23.6%) and four or more prior stays (n=64, 25.2%) (Table 1).

**Table 1:** Baseline characteristics of patients (n=254) hospitalized on the Med-Peds service line ^1 ^As of the first hospitalization for which patient was put on Med/Peds service line ^2 ^Seven (2.8%) patients did not report ethnicity IQR: interquartile range; PCP: primary care physician

Demographic Characteristics	N (%)
Age^1^, median (IQR)	26 (21, 31)
Female	147 (57.9%)
Patient reported race	
White	82 (32.3%)
Black or African American	143 (56.3%)
Multi-race/Other/Refused	29 (11.4%)
Patient reported ethnicity^2^	
Hispanic	12 (4.7%)
Non-Hispanic	235 (92.5%)
Not reported	7 (2.8%)
Distance from hospital	
Distance>15 miles	178 (70.1%)
Distance <= 15 miles	76 (29.9%)
Insurance type^1^	
None	23 (9.1%)
Private	88 (34.6%)
Public	143 (56.3%)
PCP listed in chart^1^	217 (85.4%)
Discharges from hospital in the past 24 months^1^	
0	130 (51.2%)
1-3	60 (23.6%)
4+	64 (25.2%)

TRAQ

**Figure 4:**
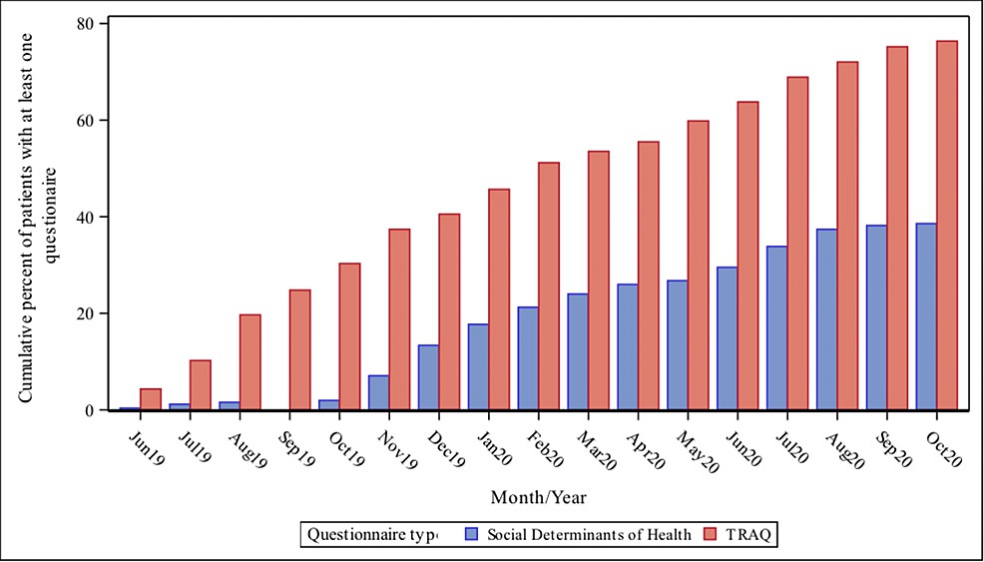
Percentage of patients with Transition Readiness Assessment Questionnaire (TRAQ) and Social Determinants of Health (SDH) questionnaire completed during a Med-Peds service line hospitalization

During the study period, 194 (76.4%) patients hospitalized on the line had at least one questionnaire administered (Figure 4). Patients who completed the TRAQ had higher numbers of previous discharges (p=0.0082) and were younger (p=0.0031) compared to those who did not complete the TRAQ (Table 2).

**Table 2:** Med-Peds service line patient characteristics by TRAQ and SDH screening status TRAQ: Transition Readiness Assessment Questionnaire; SDH: Social Determinant of Health

	Has TRAQ			Has SDH			
	No (N=60)	Yes (N=194)	p-value	No (N=131)	Yes (N=123)	p-value	
Demographic Characteristics							
Age at service line entry	28.0 (23.0, 35.5)	25.0 (21.0, 30.0)	0.0031	27.0 (21.0, 31.0)	26.0 (21.0, 30.0)	0.7911	
Age at service line entry			0.0292			0.5002	
18-22	13 (21.7%)	65 (33.5%)		42 (32.1%)	36 (29.3%)		
23-29	20 (33.3%)	76 (39.2%)		45 (34.4%)	51 (41.5%)		
30+	27 (45.0%)	53 (27.3%)		44 (33.6%)	36 (29.3%)		
Patient sex			0.9342			0.7632	
Female	35 (58.3%)	112 (57.7%)		77 (58.8%)	70 (56.9%)		
Male	25 (41.7%)	82 (42.3%)		54 (41.2%)	53 (43.1%)		
Patient race			0.1872			0.0022	
White	25 (41.7%)	57 (29.4%)		48 (36.6%)	34 (27.6%)		
Black or African American	30 (50.0%)	113 (58.2%)		61 (46.6%)	82 (66.7%)		
Multi-race/Other/Refused	5 (8.3%)	24 (12.4%)		22 (16.8%)	7 (5.7%)		
Ethnicity			0.3612			0.2352	
Hispanic	1 (1.7%)	11 (5.7%)		9 (6.9%)	3 (2.4%)		
Non-Hispanic	58 (96.7%)	177 (91.2%)		118 (90.1%)	117 (95.1%)		
Not reported	1 (1.7%)	6 (3.1%)		4 (3.1%)	3 (2.4%)		
Health/Clinical Characteristics							
Chronic Childhood Onset Disease			0.3822			0.2082	
Congenital malformation	5 (8.3%)	10 (5.2%)		7 (5.3%)	8 (6.5%)		
Type 1 diabetes	9 (15.0%)	28 (14.4%)		18 (13.7%)	19 (15.4%)		
Type 2 diabetes	3 (5.0%)	12 (6.2%)		10 (7.6%)	5 (4.1%)		
Inflammatory Bowel Disease	9 (15.0%)	18 (9.3%)		15 (11.5%)	12 (9.8%)		
Other	7 (11.7%)	18 (9.3%)		12 (9.2%)	13 (10.6%)		
Sickle cell	9 (15.0%)	48 (24.7%)		22 (16.8%)	35 (28.5%)		
Asthma	6 (10.0%)	7 (3.6%)		10 (7.6%)	3 (2.4%)		
Autoimmune disorder	4 (6.7%)	24 (12.4%)		13 (9.9%)	15 (12.2%)		
Genetic	4 (6.7%)	14 (7.2%)		12 (9.2%)	6 (4.9%)		
Neurological disorder	4 (6.7%)	15 (7.7%)		12 (9.2%)	7 (5.7%)		
Insurance type			0.9752			0.2302	
None	5 (8.3%)	18 (9.3%)		13 (9.9%)	10 (8.1%)		
Private	21 (35.0%)	67 (34.5%)		51 (38.9%)	37 (30.1%)		
Public	34 (56.7%)	109 (56.2%)		67 (51.1%)	76 (61.8%)		
Does patient have PCP?	50 (83.3%)	167 (86.1%)	0.5982	114 (87.0%)	103 (83.7%)	0.4592	
Discharges in the past 24 months			0.0082			<0.0012	
0	37 (61.7%)	93 (47.9%)		83 (63.4%)	47 (38.2%)		
1-3	17 (28.3%)	43 (22.2%)		34 (26.0%)	26 (21.1%)		
4+	6 (10.0%)	58 (29.9%)		14 (10.7%)	50 (40.7%)		

**Figure 5:**
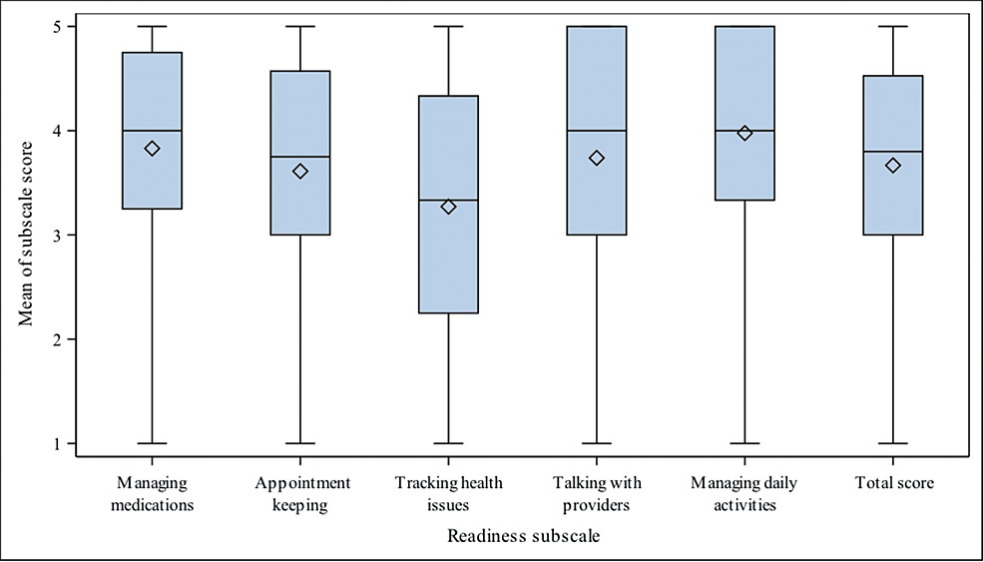
Boxplot summarizing distribution of average TRAQ 5.0 subscale summary scores Average scores in each of the five domains of the TRAQ 5.0. 1= No, I do not know how; 2= No, but I want to learn; 3= No, but I am learning to do this; 4= Yes, I have started doing this; 5= Yes, I always do this when I need to. TRAQ: Transition Readiness Assessment Questionnaire

Those who completed the TRAQ had overall median scores of 3.8 (3.0-4.5), corresponding to an answer between “No, but I’m learning to do this” and “Yes, I have started doing this” (Figure 5). The lowest median TRAQ scores were observed for the Tracking Health Issues subscale (median=3.3, interquartile range (IQR): 2.3-4.3). The highest median subscale scores were for Managing Daily Activities and Managing Medications (median 4.0), corresponding to a median response of “Yes, I have started doing this”.

SDH

The overall rate of SDH screening completion was 48.4% (n=123), with 98 patients (38.6%) having an SDH questionnaire completed during an MP line hospitalization (Table 3). The majority (n=82, 66.7%) of patients reported the presence of at least one SDH. Of those with an unmet social need, 53 (48.6%) reported financial strain, 62 (55.4%) reported food scarcity, 64 (55.2%) reported worrying about food security at least some of the time, and 54 (44.6%) reported a need for medical transportation. A larger proportion of patients who had completed SDH screening were Black (n=82, 66.7%) compared to those who did not complete the screening (n=61, 46.6%) (p=0.002). Patients who completed the screening were more likely to have been previously hospitalized (n=76, 61.8%), compared to those without the screening, of whom 36.7% (n=48) were previously hospitalized (p=0.001) (Table 2).

**Table 3:** Summary of reports of any difficulty with SDH ^1^Missing values for each question as follows: any financial strain (n=14), any food insecurity (scarcity) (n=11), any food insecurity (worry) (n=7), need for medical transportation (n=2), other transportation needs (n=3) ^2^Responded somewhat hard, hard, or very hard from options of: not hard at all, not very hard, somewhat hard, hard, very hard ^3^Responded sometimes true or often true from options of: never true, sometimes true, often true ^4^Responded yes from options of: yes, no ^5^Positive response to financial strain, food insecurity, medical or other transportation needs SDH: Social Determinants of Health

	N (%)
Patients with at least one SDH question answered during a service line hospitalization	98 (38.6%)
Patients with at least one SDH question answered	123 (48.4%)
Of those with any SDH responses^1^	(N=123)
Any financial strain?^2^	53 (48.6%)
Any food insecurity (scarcity)?^3^	62 (55.4%)
Any food insecurity (worry)?^3^	64 (55.2%)
Need for medical transportation^4^	54 (44.6%)
Other transportation needs^4^	52 (43.3%)
Any SDH indicator of hardship^5^	82 (66.7%)

Hospitalizations and care coordination

The 254 patients had 385 inpatient hospitalizations on the MP line during the study period. The median length of stay was 4.4 days (IQR: 2.6-6.8) with a mean length of stay of 6.6 days (SD 9) (Table 4). Most MP service line hospitalizations (n=316, 82.3%) were admitted from home or non-healthcare facilities and were discharged to home or self-care (n=317, 82.3%).

**Table 4:** Med-Peds service line admission-specific summaries ^1^Missing (n=1) ^2^Needs follow-up if not discharged to skilled nursing facility, rehabilitation facility, or deceased IQR: interquartile range; AMA: against medical advice

Measure	Med-Peds line hospitalizations (N=385)
Length of stay (days), median (IQR)	4.4 (2.6, 6.8)
Length of stay (days), mean (SD)	6.6 (9.0)
Length of stay	
0-<4 days	179 (46.5%)
4-<7 days	115 (29.9%)
7+ days	91 (23.6%)
Admission source^1^	
Home or non-health care facility	316 (82.3%)
Admission from outpatient facility	18 (4.7%)
Transfer from another hospital	50 (13.0%)
Discharge disposition	
Home or self-care	317 (82.3%)
Home health service	47 (12.2%)
Left AMA	15 (3.9%)
Other	6 (1.6%)
Needs follow-up after discharge^2^	348 (90.4%)
Follow-up appointment(s) scheduled 7 days following discharge	217 (62.4%)
Follow-up appointment(s) scheduled 30 days following discharge	308 (88.5%)
Follow-up appointments attended 7 days following discharge	109 (50.2%)
Follow-up appointments attended 30 days following discharge	231 (75.0%)
Patient readmitted within 30 days of discharge	79 (20.5%)

The majority (90.4%) of patients required follow-up after discharge (i.e., they were not discharged to a skilled nursing facility, rehabilitation facility, or deceased). Of those patients, 62.4% and 88.5% had a follow-up visit scheduled seven days and 30 days after discharge, respectively. The 30-day readmission rate of the MP service line patients was 20.0%, compared to 14.7% of all general medicine patients, during the study period.

## Discussion

The MP service line grouped hospitalized young adults with CCOD onto one team cared for by an MP-trained hospitalist and performed screening for transition readiness and social determinants of health. We describe a framework for redesigning care to meet the unique needs of this patient population by addressing acute medical care delivery, facilitating transitions of care, and coordinating care after discharge. We have identified a population with great medical complexity, unmet social needs, and poor self-management skills. The intersection of all of these factors may drive high readmission rates and be an essential factor in the declining health and poor outcomes seen among this population.

The MP service line criteria did not limit patients to certain chronic diseases, which yielded a very diverse patient population in terms of medical conditions. At our integrated adult and pediatric hospital, there is flexibility for patients with CCOD to be admitted to either pediatric or adult medicine services, based on individual patient needs, bed capacity, and provider availability. This allowed for seamless admission of young adults to our service line, which may not be feasible at other centers. Patients with CCOD may be cared for on pediatric services into their early 20s which likely caused an older shift in our patient population.

Despite their older age, most patients still had opportunities to improve their self-management and self-advocacy skills as evidenced by low scores on the TRAQ in multiple domains. This finding underlines the importance of ongoing efforts for pediatric providers to prepare patients for transfer to adult-oriented care, and for adult providers to continue to work on self-management skills once they are in adult care. Further, the completion rate of the TRAQ was high, which suggests that this tool may have applicability in the inpatient setting.

Despite a study reporting that only 38% of pediatric hospitals perform inpatient transition initiatives [15], we found that transition-specific interventions during hospitalization were possible, including administration of the TRAQ and providing education, instruction, and goal-setting based on responses. Similarly, although SDHs are rarely screened for during hospitalization, we were able to implement screening on our patients [16-18].

We found that our patients were largely on public insurance, had a high burden of unmet social needs, and had higher readmission rates compared to other general medicine patients. The association between these factors is supported by previously published work. For example, the presence of unmet social needs in adults and children is associated with poor health outcomes and increased healthcare utilization, including ED visits, admissions, and readmissions [19-21]. In addition, low socioeconomic status and Medicaid insurance have been associated with increased readmissions among patients with certain conditions [22].

There are several limitations to our study. The service line exists at a single site in a large academic, integrated hospital, which limits its generalizability. We also may have a higher prevalence of young adults with CCOD compared to other facilities due to the integrated pediatric hospital within a hospital model. Within our hospital medicine team, there were a large number of MP-trained physicians, which made it possible to start the line. While physicians trained in family medicine may also be considered for the treatment of patients across the age spectrum, our program consists only of internal medicine and MP-trained physicians.

Patients with repeated admissions had discontinuity with the line, which detracted from the potential benefits of longitudinal inpatient care. Census and team capacities forced eligible patients to be admitted to other general medicine teams. Conversely, the service line routinely cared for other general medicine patients not meeting the inclusion criteria in an effort to balance the overall general medicine census. These patients were excluded from the interventions and our data analysis.

The assignment of patients to the MP service line was based on inclusion criteria and the triage hospitalist’s own judgment, which may have introduced selection bias. Bias may also have influenced the assignment of CCOD ascertained by chart review; however, prior work to assess the accuracy of diagnosis codes suggests that these depend on definitions used, and that chart review by a clinician may be used as a gold standard [23].

The TRAQ has its own limitations, including that scores are self-reported and therefore may have bias. The tool has only been validated in patients seen in the outpatient setting [12]. SDH screening completion during the hospitalization was low. Therefore, we analyzed the responses to SDH questions regardless of whether they were performed during the hospitalization. The finding that patients who received the SDH screening were more likely Black may highlight bias by the team that Black patients were more likely to have unmet social needs, or due to low patient engagement of the questions amid a more diverse patient population.

Adherence to post-discharge appointments could not easily be accurately assessed given the lack of data for appointments outside of the health system network. Given the inverse association between adherence to care and readmission rate, particularly among patients with sickle cell disease [24], this may be valuable to investigate in the future. Finally, we report whether the patient had a primary care provider identified, which may not reflect their receipt of routine and preventative services.

## Conclusions

Assigning hospitalized young adults with CCOD to a dedicated team allows for specific interventions to address challenges seen within this population, such as transition skill assessment, identification of social needs, and coordination of care with outpatient providers. Physicians trained in Internal Medicine and Pediatrics are uniquely skilled to address these care needs, and hospitals should consider redesigning teams to match providers with this skillset with these young adult patients.

Future steps include building on resources to support patients post-discharge like care management, community resources to help meet social needs, peer mentorship, and mental health access. Partnerships with specialists to coordinate inpatient and outpatient care may prove to be effective ways to reduce gaps in care post-discharge, while also encouraging patient engagement with their medical team after discharge. Future research is needed to understand the impact of the service line interventions on patient outcomes, utilization, and experience, as well as physician satisfaction.
